# Prognostic Value of Total Bilirubin in Patients With ST-Segment Elevation Acute Myocardial Infarction Undergoing Primary Coronary Intervention

**DOI:** 10.3389/fcvm.2020.615254

**Published:** 2020-12-17

**Authors:** Xiaoxiao Zhao, Ying Wang, Chen Liu, Peng Zhou, Zhaoxue Sheng, Jiannan Li, Jinying Zhou, Runzhen Chen, Yi Chen, Hanjun Zhao, Hongbing Yan

**Affiliations:** ^1^Department of Cardiology, National Center for Cardiovascular Diseases, Peking Union Medical College & Chinese Academy of Medical Sciences, Fuwai Hospital, Beijing, China; ^2^Fuwai Hospital Chinese Academy of Medical Sciences, Shenzhen, China

**Keywords:** primary percutaneous coronary intervention, ST-segment elevation myocardial infarction, coronary artery disease, total bilirubin-based score model, total bilirubin

## Abstract

**Background:** Bilirubin, a natural product of heme catabolism, has antioxidant and anti-inflammatory activities and is inversely associated with stable coronary artery disease. However, the relationship between the bilirubin levels and long-term outcomes in patients with ST-segment elevation myocardial infarction (STEMI) who underwent primary percutaneous coronary intervention (PPCI) remains unknown. This study aimed to establish a score model based on bilirubin for predicting major adverse cardiovascular events (MACEs) and stratify patients to the level of care.

**Methods and Results:** Data of 4,151 consecutive patients with STEMI who underwent PPCI were evaluated, and 3,708 cases were analyzed. The total bilirubin (TBil) levels were measured during admission, and the study population was divided into two groups. The high TBil group (*n* = 143) comprised patients who had a TBil level of ≥22 μmmol/L, and the low TBil group (*n* = 3,565) comprised patients who had a TBil level of <22 μmmol/L. The median follow-up period was 754 days (2.066 years). The MACE was significantly lower in the high TBil group than in the low TBil group (3.5% vs. 11.0%, *p* = 0.001). In the multivariate Cox regression analysis, a significant association was noted between the TBil levels and adjusted risk of MACE (hazard ratio, 0.279; 95% confidence interval, 0.088–0.877; *p* = 0.029). A prediction score model composed of TBil, age, hypertension history, and other eight variables was developed, with scores ranging from 0 to 500. The scores categorized patients into low-, medium-, and high-risk categories. The cumulative survival rate was significantly higher in the low-risk group than in the medium- and high-risk groups for MACE, all-cause death, cardiac death, recurrent myocardial infarction, and ischemic stroke (*p* < 0.001, *p* < 0.001, *p* < 0.001, *p* = 0.030, and *p* = 0.001, respectively). The area under the curve of the TBil score was 0.768; this was significantly greater in the pairwise comparison with the Global Registry of Acute Coronary Events score (*p* = 0.0012).

**Conclusion:** The new prediction score model based on TBil could be used in clinical practice to support risk stratification as recommended in the clinical guidelines.

## What is Already Known About This Topic?

Most studies on total bilirubin (TBil) and coronary artery disease (CAD) have focused on stable CAD. Few studies have focused on the relationship between the serum TBil levels and short- and long-term clinical outcomes.

## What Does This Article Add?

A high TBil level is independently associated with major adverse cardiovascular event (MACE) in patients with ST-segment elevation myocardial infarction (STEMI) undergoing primary percutaneous coronary intervention (PPCI) at the median 2 year follow-up period. The TBil-based score model can be used to predict MACE in patients with STEMI undergoing PPCI.

## Introduction

Primary percutaneous coronary intervention (PPCI) has been a widely authoritative treatment method for subjects with ST-segment elevation myocardial infarction (STEMI) (1). Angiographic and ultrasound results are well-known to be associated with mortality and morbidity in acute STEMI (2). Early stage risk management and stratification are critical for successful initial management of patients with STEMI (3). As a natural product of heme catabolism, serum bilirubin is a potent antioxidant at oxygen concentration under physiological conditions (4). Several epidemiologic researches have demonstrated that serum total bilirubin (TBil) is inversely correlated with the incidence of metabolic syndrome, diabetes mellitus, and hypertension (5–7). Most studies on TBil and coronary artery disease (CAD) have focused on stable CAD. However, few studies have focused on the relationship between the serum TBil levels during acute stress and short- and long-term clinical outcomes.

Herein, we aimed to investigate whether the TBil level measured during admission is a prognostic indicator for major adverse cardiovascular event (MACE) in patients with STEMI who underwent PPCI and, if so, identify independent risk factors in these patients. Specifically, this study aimed to establish a score model for predicting MACE based on TBil.

## Methods

### Subjects

This single-center, retrospective, observational cohort study analyzed data from Fuwai Hospital in Beijing, China, from January 2010 to July 2018. A total of 3,708 subjects were enrolled (97 patients were lost to follow-up, and 346 had missing bilirubin data) from 4,151 consecutive patients with STEMI. We referred to the guidelines (8, 9) of acute STEMI fulfilling the criteria. Blood samples for bilirubin measurement were obtained on admission and after PPCI. Finally, 3,708 patients were included in the analysis.

The research protocol was reviewed and approved (reference number 2016-I2M-1–009) by the Ethics Committee of Fuwai Hospital, and all subjects provided informed consent.

### Definitions

In this study, STEMI was defined based on symptoms (i.e., continuous chest pain lasting >30 min), electrocardiography (ECG) finding (i.e., ST-segment elevation > 0.1 mV on an 18-lead ECG), and laboratory testing (i.e., increasing cardiac troponin I level) (10). The primary endpoint was a composite of all-cause mortality, cardiac cause death, recurrent myocardial infarction (MI), and ischemic stroke. MACE was composite of all-cause mortality, recurrent MI, and ischemic stroke.

### Statistical Analysis

The study population was divided into tertiles according to the study cohort. The high TBil group (*n* = 143) was composed of patients who had a TBil value in the third tertile (>22 μmmol/L), and the low TBil group (*n* = 3,565) was composed of patients who had a TBil value in the lower two tertiles (≤ 22 μmmol/L). We used two-tailed Student's *t*-test to compare parametric values and the chi-square test to compare categorical variables between the two groups. The multivariate logistic regression analysis (backward stepwise) was performed to identify the association between the variables and incidence of MACE. The Kaplan–Meier method (the Breslow generalized Wilcoxon test was used to assess differences) was used to compare the time-to-event curves between different groups; *p* < 0.05 was considered statistically significant. The regression coefficients were converted to integer risk scores, and the final score was the sum of these values. The diagnostic value of the new score model was estimated using the area under the curve (AUC). The accuracy of the TBil-based and Global Registry of Acute Coronary Events (GRACE) scores in predicting MACE, death, recurrent MI, and stroke was assessed using the receiver-operating characteristic (ROC) curves and compared with the MedCalc software for Windows, version 18.2.1, using a non-parametric test developed. The corresponding nomogram prediction model was drawn according to the regression coefficient of the selected independent variables. The model is derived using the formula: probability of event at time, t = S0(t)exp(β1x1+β2x2…) where S0(t) is called the baseline survival function estimated from the data, x are the reported values of the covariates. β is the regression coefficients. Regression coefficients are used to construct the variable axes in the nomogram and S0 is used in the translation from total points to predicted probability. The main statistical analysis software used in this study was the R language version I 386 3.6.2. Other statistical analyses were performed using SPSS version 20.0 (SPSS, Chicago, IL, USA).

## Results

### Patient Demographics

Of the 3,708 patients, there were 3,565 in the low TBil group and 143 in the high TBil group. The baseline characteristics of the two groups are summarized in Table 1. The numbers of patients with atrial fibrillation (*p* = 0.026) and stent implantation (*p* = 0.016) were higher in the high TBil group than in the low TBil group. Patients in the high TBil group had a lower incidence of MACE (*p* = 0.001), death (*p* = 0.026), and recurrent MI (*p* = 0.039). The bivariate correlation analysis between the serum TBil levels and laboratory examinations, cardiovascular disease risk factors, discharge medication regimen, and endpoint events is shown in Table 1. The serum TBil level was inversely correlated with age (correlation coefficient = −0.060, *p* < 0.001), systolic blood pressure (−0.045, *p* = 0.007), hypertension (−0.043, *p* = 0.009), diabetes mellitus (−0.049, *p* = 0.003), history of PCI (−0.038, *p* = 0.020), incidence of MACE (−0.104, *p* < 0.001), death (−0.094, *p* < 0.001), cardiac death (−0.076, *p* < 0.001), and ischemic stroke (−0.051, *p* < 0.001).

**Table 1:** Association between the bilirubin and laboratory and angiographic procedural-characteristics of patients.

**Variables**	**Serum TBil (*****n*** **=** **3,708)**		**Low Serum TBil (*n* = 3,565)**	**High Serum TBil (*n* = 143)**	***p*-value**
	**Correlation coefficient**	***P*-value**			
**LABORATORY EXAMINATIONS**					
Age, years	−0.060	<0.001^*^	59.23 ± 0.199	56.58 ± 0.986	0.009^*^
Male	0.069	<0.001^*^	2,778(77.9%)	131(91.6%)	<0.001^*^
Peak c-TnI mg/dl	0.015	0.432	3.59 ± 0.215	3.86 ± 1.058	0.797
HDL-cholesterol (mg/dl)	0.001	0.950	1.69 ± 0.019	1.63 ± 0.076	0.484
LDL-cholesterol (mg/dl)	0.017	0.312	2.74 ± 0.016	2.74 ± 0.076	0.957
Triglycerides (mg/dl)	−0.019	0.242	1.05 ± 0.005	1.05 ± 0.022	0.852
hs-CRP	−0.029	0.085	7.61 ± 0.084	7.40 ± 0.422	0.612
SCr	−0.017	0.289	82.37 ± 0.418	83.99 ± 2.642	0.451
eGFR(MDRD formulation)	0.032	0.052	89.55 ± 1.409	96.70 ± 10.233	0.331
D-dimer	−0.021	0.679	0.67 ± 0.030	0.66 ± 0.135	0.946
Lpa	−0.007	0.679	268.31 ± 4.159	238.07 ± 19.651	0.153
EF at admission	−0.004	0.800	53.91 ± 0.238	53.766 ± 0.641	0.902
Systolic pressure	−0.045	0.007^*^	124.26 ± 0.329	122.91 ± 1379	0.414
Diastolic blood pressure	0.019	0.251	70.79 ± 0.278	72.69 ± 1.538	0.180
**RISK FACTORS**					
Hypertension [% (*n*)]	−0.043	0.009^*^	2,187(61.3%)	84(3.58.7%)	0.293
Hyperlipidemia [% (*n*)]	0.045	0.010^*^	2,919(92.1%)	111(93.3%)	0.407
Smoking [% (*n*)]	0.025	0.158	2,101(65.8%)	86(72.3%)	0.084
Diabetes [% (*n*)]	−0.049	0.003^*^	1,168(32.8%)	44(30.8%)	0.345
History of PCI [% (*n*)]	−0.038	0.020^*^	491(13.8%)	14(9.8%)	0.105
History of CABG [% (*n*)]	−0.020	0.235	41(1.2%)	0(0.0%)	0.198
Atrial fibrillation [% (*n*)]	−0.025	0.132	215(6.0%)	3(2.1%)	0.026^*^
Chronic kidney disease [% (*n*)]	−0.021	0.197	283(7.9%)	6(4.2%)	0.061
**ANGIOGRAPHIC FINDINGS PRE-ppci AND PROCEDURAL DATAS**					
LM lesion [% (*n*)]	−0.021	0.210	238 (6.8%)	7 (4.9%)	0.248
Diameter of the target lesion	0.037	0.025^*^	3.18 ± 0.013	3.26 ± 0.063	0.198
Length of the target lesion	0.010	0.555	27.16 ± 0.254	27.23 ± 1.204	0.955
Degree of lesion stenosis	0.004	0.789	37.48 ± 0.656	35.59 ± 3.230	0.571
Bifurcation lesions	−0.013	0.441	1,195 (34.2%)	50 (35.2%)	0.431
PTCA	0.037	0.037^*^	3,065 (87.6%)	128 (90.1%)	0.228
Thrombus aspiration	0.017	0.304	1,476 (42.2%)	68 (47.9%)	0.105
Stent implantation	0.053	0.001^*^	3,096 (88.5%)	134 (88.8%)	0.016^*^
The use of IABP	−0.023	0.171	343 (9.8%)	14 (9.9%)	0.535
**DISCHARGE MEDICATION REGIMEN**					
Statin [% (*n*)]	0.042	0.011^*^	3,267 (93.4%)	140 (98.6%)	0.004^*^
Aspirin [% (*n*)]	0.039	0.019^*^	3,464 (99.1%)	142 (100%)	0.267
Clopidogrel [% (*n*)]	−0.149	<0.001^*^	2,749 (78.6%)	91 (64.1%)	<0.001^*^
Ticagrelor [% (*n*)]	0.156	<0.001^*^	721 (20.7%)	51 (35.9%)	<0.001^*^
ACEI [% (*n*)]	0.045	0.006^*^	2,161 (61.8%)	96 (67.6%)	0.094
ARB [% (*n*)]	−0.032	0.051	311 (8.9%)	9 (6.3%)	0.185
Beta-Blockers [% (*n*)]	0.018	0.285	3,062 (87.6%)	124 (87.3%)	0.506
Diuretic [% (*n*)]	−0.002	0.909	1,018 (29.1%)	52 (36.6%)	0.035^*^
Spironolactone [% (*n*)]	0.000	0.978	755 (22.2%)	34 (23.9%)	0.340
**ENDPOINT EVENTS**					
MACE [% (*n*)]	−0.104	<0.001^*^	392 (11.0%)	5 (3.5%)	0.001
Death [% (*n*)]	−0.094	<0.001^*^	215 (6.0%)	3 (2.1%)	0.026^*^
CV death [% (*n*)]	−0.076	<0.001^*^	143 (4.0%)	2 (1.4%)	0.075
Recurrence MI	−0.023	0.160	126 (3.5%)	1 (0.7%)	0.039^*^
Revascularization [% (*n*)]	−0.009	0.592	526 (14.8%)	18 (12.6%)	0.281
Ischemic stroke [% (*n*)]	−0.051	0.002^*^	67 (1.9%)	1 (0.7%)	0.254
Hemorrhagic stroke [% (*n*)]	−0.008	0.619	13 (0.4%)	1 (0.7%)	0.424

*Data are expressed as mean ± SE (Standard error) for normally distributed data and n (%) for categorical variables*.

*cTnI, cardiac troponin I; HDL, high-density lipoprotein; LDL, low-density lipoprotein; CRP, C-reactive protein; ALT, alanine aminotransferase; AST, aspartate aminotransferase; TBil, total bilirubin; D-BIL, direct bilirubin; ApoA, apolipoprotein A; ApoB, apolipoprotein B; LAD, left atrial diameter; IVSd, interventricular septal thickness diameter; LVEDV, left ventricular end systolic volume; LVPWs, left ventricular posterior wall thickness; EF, ejection fraction; ACEI, angiotensin-converting enzyme inhibitor; ARB, angiotensin receptor blocker; MACE, major adverse cardiovascular events; CV death, cardiovascular death*.

* *p < 0.05*.

### Factors Associated With MACE

Table 2 shows the results of the univariate and multivariate Cox regression analyses. The univariate Cox regression analysis revealed that age > 60 years, male sex, STEMI, history of hypertension, diabetes mellitus, atrial fibrillation, previous coronary artery bypass grafting, previous PCI, chronic kidney disease, smoking, ejection fraction (EF), high-density lipoprotein-cholesterol <0.7 mg/dL, and other 30 variables (Table 2) were found to be significant risk factors for the incidence of MACE. The multivariate Cox regression analysis showed that age > 60 years (*p* < 0.001), history of hypertension (*p* = 0.012), history of chronic kidney disease (*p* < 0.001), EF (*p* = 0.006), TBil (*p* = 0.029), stent implantation (*p* = 0.001), Killip classification (*p* < 0.000), use of tirofiban during PCI (*p* = 0.016), coronary revascularization at admission (*p* = 0.003), and use of angiotensin-converting enzyme inhibitors (ACEIs) (*p* = 0.031) and beta-blockers (*p* < 0.001) remained independent risk factors associated with a high risk of mortality.

**Table 2:** Factors associated with MACE in patients with STEMI following PPCI.

**Variables**	**Univariate Cox regression**	***P*-value**	**Multivariate Cox regression**	***P*-value**
	**HR (95% CI)**		**HR (95% CI)**	
Age > 60 y	2.570 (2.083–3.171)	<0.001^*^	1.903 (1.512–2.395)	<0.001^*^
Male	0.565 (0.456–0.701)	<0.001^*^	–	–
STEMI	0.732 (0.541–0.992)	0.045^*^	–	–
Hypertension	1.495 (1.207–1.851)	<0.001^*^	1.350 (1.068–1.705)	0.012^*^
Diabetes mellitus	1.440 (1.177,1.761)	<0.001^*^	–	–
Atrial fibrillation	2.660 (1.987–3.561)	<0.001^*^	–	–
Prior CABG	2.756 (1.422–5.342)	0.003^*^	–	–
Prior PCI	1.412 (1.965–1.872)	0.017^*^	–	–
Chronic kidney disease	3.181 (2.449–4.133)	<0.001^*^	1.780 (1.318–2.405)	<0.001^*^
Smoking	0.681 (0.556–0.834)	<0.001^*^	–	–
EF > 50%	1.757 (1.426–2.167)	<0.001^*^	1.378 (1.096–1.733)	0.006^*^
HDL <0.7 (mg/dl)	1.367 (1.005–1.859)	0.047^*^	–	–
LDL > 3.12 (mg/dl)	0.951 (0.763–1.186)	0.655	–	–
Triglycerides > 1.7 (mg/dl)	1.200 (0.704–2.046)	0.503	–	–
LPA > 300 (g/L)	1.165 (0.946–1.436)	0.151	–	–
hs-CRP > 10	1.379 (1.126–1.689)	0.002^*^	–	–
D-dimer > 0.2 mg/L	1.358 (1.088–1.694)	0.007^*^	–	–
eGFR > 90	0.392 (0.315–0.490)	<0.001^*^	–	–
Creatinine > 133 umol/L	3.546 (2.470–5.091)	<0.001^*^	–	–
Total bilirubin > 22 ummol/L	0.413 (0.171–0.998)	0.049^*^	0.279 (0.088–0.877)	0.029^*^
TIMI flow pre-PCI			–	–
0	Ref	0.970	–	–
1	1.024 (0.608–1.724)	0.920	–	–
2	0.946 (0.656–1.366)	0.769	–	–
3	1.049 (0.79701.379)	0.733	–	–
Bifurcation lesions	0.899 (0.725–1.116)	0.336	–	–
PTCA	1.209 (0.894–1.634)	0.218	–	–
Thrombus aspiration	0.855 (0.693–1.055)	0.144	–	–
Stent implantation	0.587 (0.444–0.776)	<0.001^*^	0.683 (0.509–0.917)	0.011^*^
KILLIP	Ref	<0.001^*^	Ref	<0.001^*^
KILLIP II	1.639 (1.222–2.199)	0.001^*^	1.228 (0.895–1.684)	0.204
KILLIP III	3.053 (1.816–5.134)	<0.001^*^	1.671 (0.943–2.961)	0.079
KILLIP IV	8.669 (6.437–11.674)	<0.001^*^	4.349 (2.988–6.331)	<0.001^*^
LM lesion	2.292 (1.682–3.121)	<0.001^*^	1.442 (1.024–2.030)	0.036^*^
Number of coronary artery lesions			–	–
1	Ref	<0.001^*^	–	–
2	1.656 (1.199–2.288)	0.002^*^	–	–
3	2.358 (1.751–3.174)	<0.001^*^	–	–
Use of tirofiban during PCI	0.642 (0.464–0.889)	0.008^*^	0.666 (0.478–0.927)	0.016^*^
Coronary revascularization at admission	0.572 (0.461–0.711)	<0.001^*^	0.710 (0.567–0.889)	0.003^*^
Statin	1.243 (0.815–1.897)	0.312	–	–
Aspirin	0.413 (0.205–0.832)	0.013^*^	–	–
Clopidogrel	1.121 (0.771–1.631)	0.549	–	–
Ticagrelor	0.983 (0.666–1.489)	0.983	–	–
ACEI	0.607 (0.496–0.744)	<0.001^*^	0.788 (0.634–0.978)	0.031^*^
Beta-Blockers	0.543 (0.419–0.705)	<0.001^*^	0.565 (0.431–0.741)	<0.001^*^

*HDL, high-density lipoprotein; LDL, low-density lipoprotein; CRP, C-reactive protein; EF, ejection fraction; ACEI, angiotensin-converting enzyme inhibitor; MACE, major adverse cardiovascular events; PCI, percutaneous coronary intervention; PTCA, Percutaneous transluminal coronary angioplasty; CABG, coronary artery bypass graft; TIMI, thrombolysis in myocardial infarction; y, year*.

* *p < 0.05*.

### Prediction Score Model

**Figure 1:**
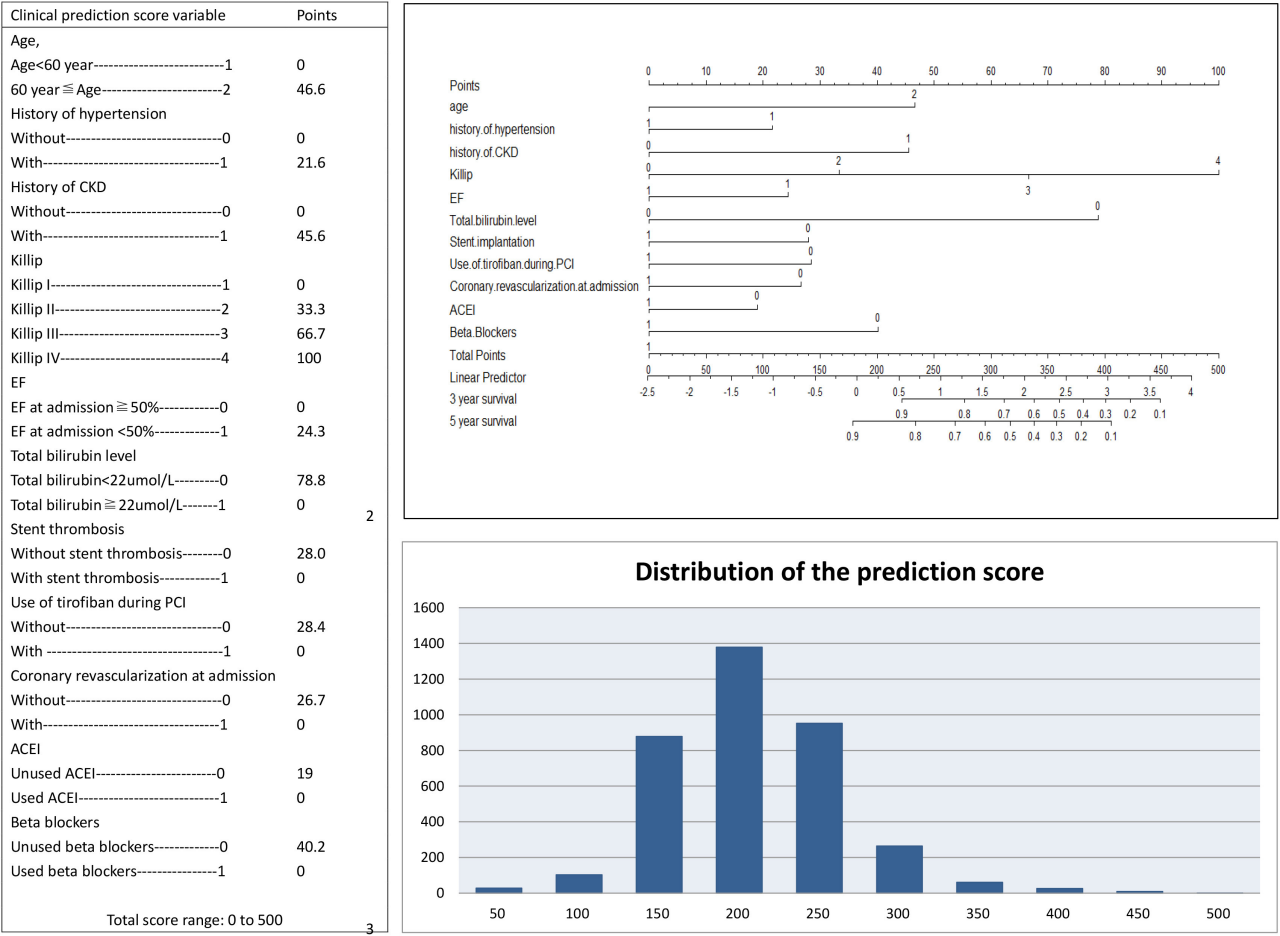
Total bilirubin based Risk Score for MACE.

**Figure 2:**
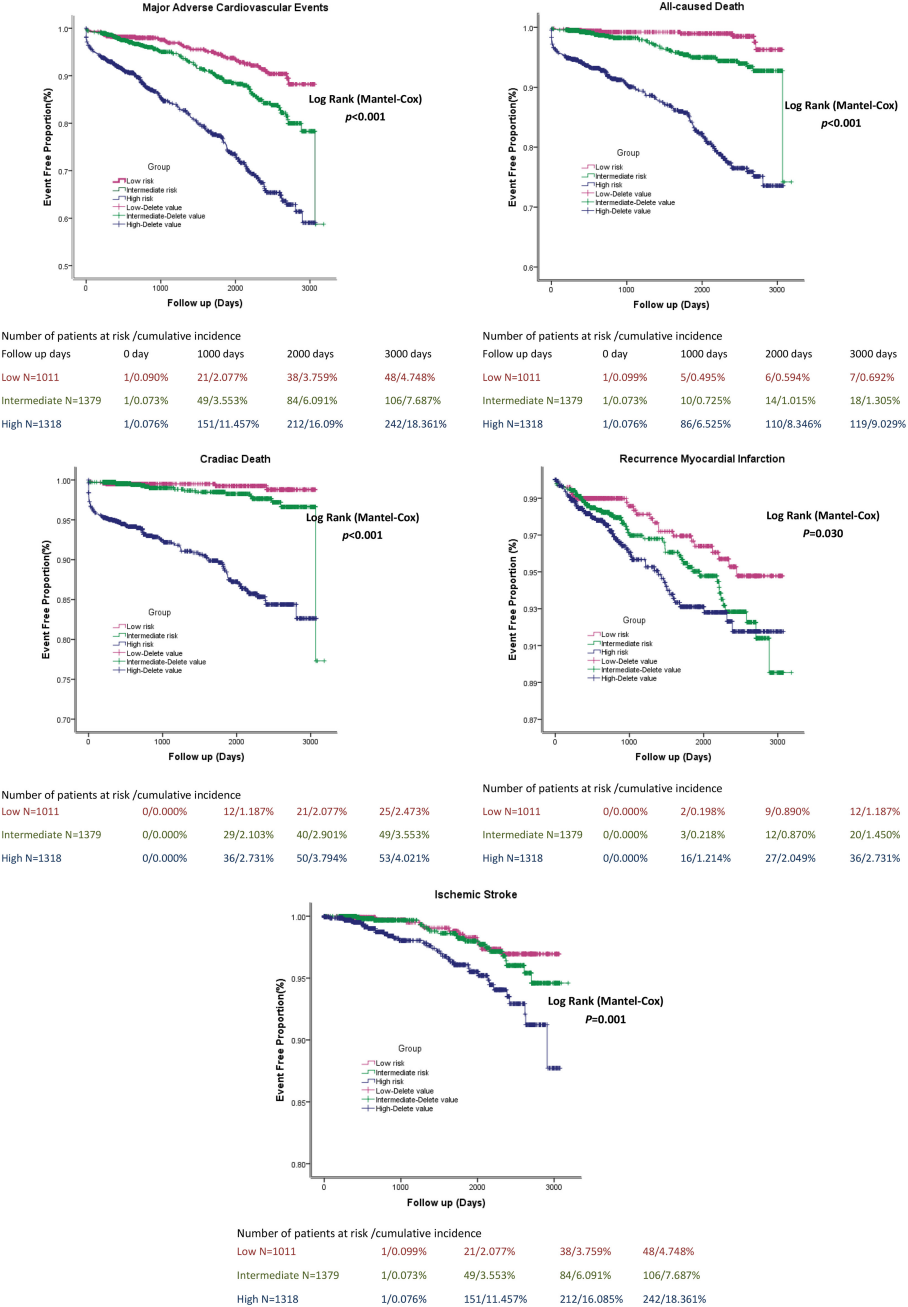
The cumulative survival rate of patients in stratified Bilirubin based-score model groups.

A simplified risk score was generated to predict MACEs based on TBil. Scoring was performed during hospitalization to predict long-term events by physicians. In the nomogram prediction model, the values of the selected variables can correspond to the scores on the integral line at the top of the nomogram through the projection of the vertical line, and the total score can be obtained by adding the scores corresponding to the values of each variable. The cumulative occurrence probability of MACEs at 3 and 5 years can be obtained from the total score on the prediction line at the bottom of the nomogram. The scores, which ranged from 0 to 500 points, were assigned as follows: age <60 years, 0; age ≥ 60 years, 46.6; without history of hypertension, 0; with history of hypertension, 21.6; without history of CKD, 0; with history of CKD, 45.6; Killip I, 0; Killip II, 33.3; Killip III, 66.7; Killip IV, 100; EF at admission ≥ 50%, 0; EF at admission <50%, 24.3; TBil <22 μmol/L, 78.8; TBil ≥ 22 μmol/L, 0; without stent thrombosis, 28.0; with stent thrombosis, 0; without the use of tirofiban during PCI, 28.4; with the use of tirofiban during PCI, 0; without coronary revascularization at admission, 26.7; with coronary revascularization at admission, 0; without the use of ACEI, 19; with the use of ACEI, 0; without the use of beta-blockers, 40.2; and with the use of beta-blockers, 0 (Figure 1). The elements of the clinical prediction score model and distribution of scores among patients who underwent PPCI are shown in Figure 1. Further analysis was performed to evaluate the influence of each risk factor on the cumulative survival rate. The cumulative survival rates of MACE (*p* < 0.001), all-cause death (*p* < 0.001), cardiac death (*p* < 0.001), recurrent MI (*p* = 0.030), and ischemic stroke (*p* < 0.001) were significantly higher in the low-risk group than in the medium- and high-risk groups (Figure 2).

**Figure 3:**
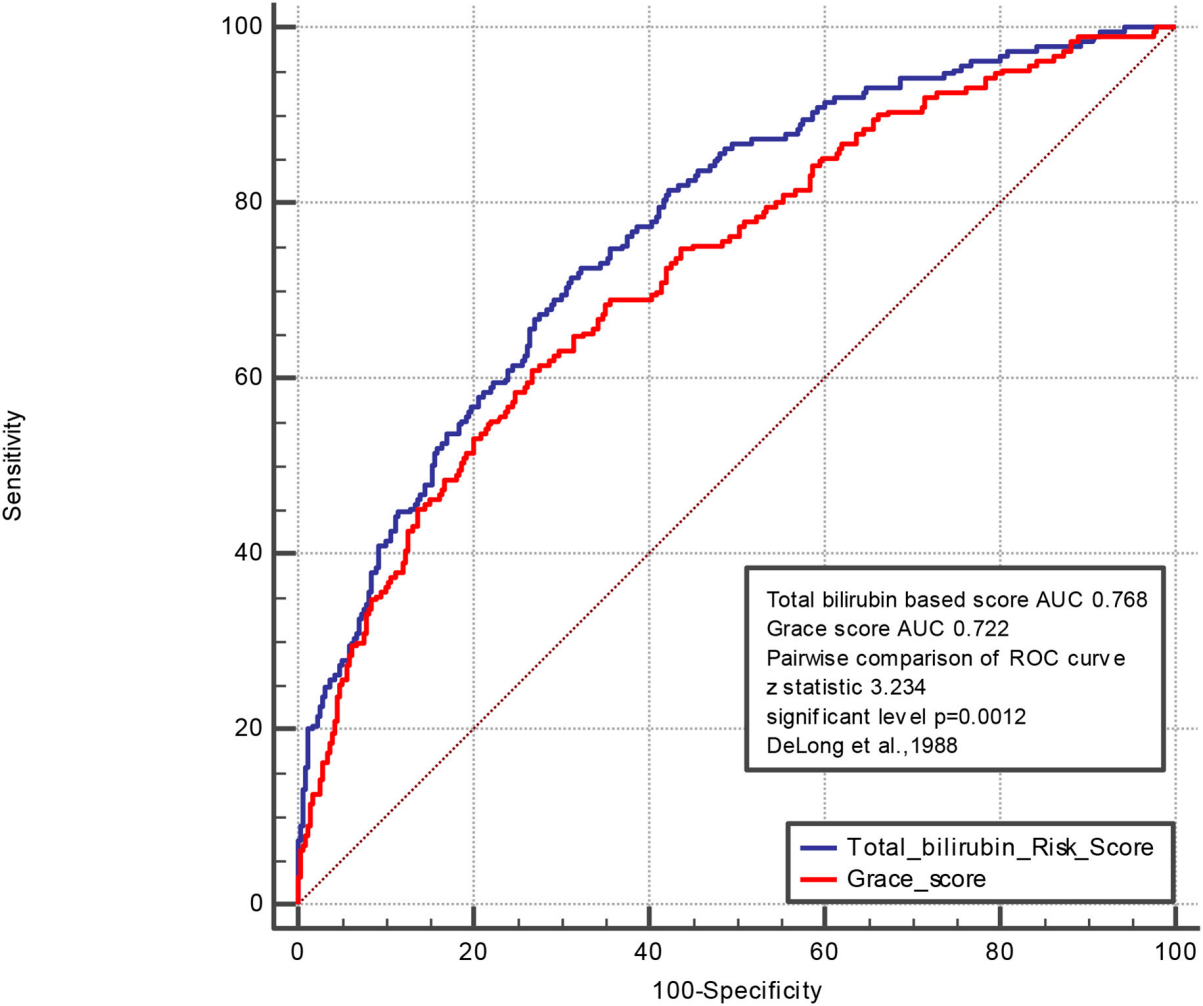
Pairwise comparison of ROC curve of Total bilirubin based score and Grace score. The areas under the curve of the TBil-based score and GRACE score were 0.768 and 0.722, respectively (*p* = 0.0012). We control the variables including age, history of diabetes mullets, history of atrial fibrillation, hyperlipidemia, history of CABG, history of PCI, current smoking, the serum level of LDL, CRP, Cr, Egfr, D-dimer, LPA, HDL, TG, heart rate, systolic blood pressure, diastolic blood pressure, height, weight, the use of ARB, statin, aldactone, diuretic, left main coronary artery lesion, percutaneous transluminal coronary angioplasty, bifurcation lesion, the peak level of troponin.

A pairwise comparison of the ROC curve of the TBil-based and GRACE scores is shown in Figure 3. The AUCs of the TBil-based and GRACE scores were 0.768 and 0.722, respectively (*p* = 0.0012).

## Discussion

In this study involving 3,708 real-world patients, we reported that the TBil-based score model can be used to predict MACEs in patients with STEMI undergoing PPCI. It is crucial to individualize and be precise in the management and assessment of patients with acute coronary syndrome while considering the disease type and stage. A prediction score model, with scores ranging from 0 to 500, composed of TBil, age, hypertension history, and other eight variables was developed. The scores categorized patients into low-, medium-, and high-risk categories. The cumulative survival rate was significantly higher in the low-risk group than in the medium- and high-risk groups for MACE, all-cause death, cardiac death, recurrent MI, and ischemic stroke. In summary, we developed a prediction score model to evaluate the 3 and 5 year risk probability for patients with MI who underwent PPCI based on the measurement of TBil, and this model can be used by primary healthcare professionals and specialists to improve the risk assessment in the long-term follow-up.

### Mechanism of Bilirubin Against Atherosclerosis

STEMI has contributed to worldwide increases in the morbidity and mortality rates of CAD. The management of patients with STEMI early after symptom onset is extremely significant (11, 12). One of the major findings of the study by Celik et al. (13) is that the serum bilirubin levels, a sign of heme oxygenase-1 (HO-1) enzyme activity, were independently associated with the development of the coronary no-reflow phenomenon in PPCI. This phenomenon is one of the major adverse events in patients with STEMI undergoing PPCI. It is regarded that lower mortality among patients with STEMI and improved ventricular performance are associated with the rapid restoration of infarct-related artery flow (14). These factors result in non-reflow, including the accumulation of microvascular leukocytes, platelet plugging, increased levels of reactive oxygen species, ischemic endothelial damage, and complex interactions between leukocytes and platelets induced by the inflammatory process (15). However, in the univariate logistic regression analysis, no independent association was found between the serum bilirubin levels and development of the coronary no-reflow phenomenon in PPCI. Bilirubin has antioxidative, anti-inflammatory, and cytoprotective properties. The administration of bilirubin inhibits inflammation induced by intraportal islet transplantation. Zhu et al. (16) showed that bilirubin inhibited the production of tumor necrosis factor-α, interleukin-1β, soluble intercellular adhesion molecule-1, and monocyte chemoattractant protein-1. Several studies (17, 18) have reported that bilirubin is a potent antioxidant and plays an important role in acute and severe hypoxic injuries, inhibiting nitrous oxide expression and nitric oxide production in response to endotoxin in rats. HO, a stress-induced enzyme, is responsible for the degradation of heme groups and catalyzes pro-oxidant heme to break into biliverdin, free iron, and carbon monoxide. The released iron is sequestered to ferritin, and biliverdin is then quickly converted to bilirubin by biliverdin reductase (19). Bilirubin, as an antioxidant, prevents the oxidation of low-density lipoprotein-cholesterol (LDL-C) and plays as a mediator to inhibit anti-inflammatory processes and as a scavenger for oxygen species to resist the cytotoxic effects derived from free heme released by extracellular hemoglobin, especially accompanied by oxidative stress and inflammation (20, 21). The released heme, which is associated with the pathogenesis of atherosclerosis, can express cellular adhesion molecules and catalyze the oxidation of LDL-C (22–24). HO-1 and HO-2 are isozymes of HO. The enzyme activity of HO-1 upregulation contributes to cytoprotection in response to MI, restricts tissue damage, and regulates post-MI remodeling (25). HO-2 overexpression has also been shown to protect the brain against ischemic damage and oxidative stress (26). The testing of biomarkers that present oxidative stress index including oxidized LDL (ox-LDL), superoxide dismutase, glutamine synthase, and reduced glutathione was not conducted routinely in Fuwai Hospital. High-sensitivity C-reactive protein, which triggers monocyte–macrophage activation, binds with ox-LDL to impair vasodilation and prompts endothelial dysfunction through inflammation and via an oxidative stress pathway plays a key role in the progress of plaque formation and is considered proatherogenic (27, 28). However, in this study, no significant difference was found between the low serum TBil and high serum TBil groups (*p* = 0.612).

Previous studies suggested that oxidized stress may cause endothelial dysfunction through the increased production of reactive oxygen species (29, 30). The balance of the oxidative and antioxidative systems plays a crucial part in vascular health. A tremendous number of vascular events was induced by the increased levels of reactive oxygen species produced in the vessel wall (31). Lipid peroxidation plays an important role in the initiation and progression of atherothrombosis in the vascular wall. Several studies (32, 33) have observed that bilirubin and its precursor, biliverdin, can inhibit the oxidation of LDL-C, which plays a key role in atherogenesis and can remove reactive oxygen radicals. Nakayama et al. (34) found that prostacyclin had an antioxidant function in the early atherogenesis period through the induction of HO-1.

Mehmet Gul et al. (11) suggested that TBil is widely available to clinicians as a biochemistry parameter and a powerful prognostic factor for patients undergoing PPCI for STEMI. Xu et al. (35) reported that the high levels of TBil and direct bilirubin (DB), but not indirect bilirubin (IDB), were associated with an increased risk of MACE in Chinese patients with acute coronary syndrome, and the prognostic value of DB was superior to that of TBil or IDB. Subsequent studies (36) have suggested that bilirubin can be used for risk stratification in patients with STEMI.

### Contribution and Benefits of the Newly Established Risk Score Model Based on TBil

In this study, we established a risk score model based on TBil to predict MACE in patients with STEMI who underwent PPCI. This model is not only accurate and sensitive to predict 2 year MACE but also simple and easy to apply in clinical practice, as it is composed of three routinely available clinical and laboratory parameters. Patients in different risk groups had distinctly varying risks of MACE. Predicting the risk of death could allow clinicians to pay more attention to the high-risk group and provide them more appropriate medical care; thus, healthcare resources could be allocated and utilized more efficiently. In most studies, age is believed to be an independent factor associated with prognosis, and it is an important parameter in various score models (37). In this study, patients aged <60 years had a longer median survival time than those aged >60 years. Several studies have compared the potential of the Killip classification in predicting the prognosis of patients with STEMI (38–40). This study confirmed that the Killip classification was an independent risk factor in patients with STEMI who underwent PPCI. In addition, we observed that the non-use of tirofiban during PCI has contributed 28.4 points to the final prediction score. Tirofiban, a non-peptide tyrosine derivate, acts as a reversible inhibitor of the glycoprotein IIb/IIIa receptor (41) and was recommended by the 2009 European Society of Cardiology STEMI guidelines as a comedication to standard dual antiplatelet therapy. Previous studies have proven that tirofiban improves clinical outcome by decreasing the incidence of MACE (42) among patients undergoing PCI. In our study, patients using tirofiban received a more complex catheter intervention, had increased cardiac enzymes, or showed enlarged infarct sizes. Furthermore, we conducted a pairwise comparison of the ROC curve of the TBil-based and GRACE scores (the AUCs were 0.768 and 0.722, respectively; *p* = 0.0012). The bilirubin prediction model could comprehensively evaluate long-term prognosis in terms of oxidative stress and inflammatory response, which are necessary to improve clinical outcomes, subsequent management, and risk prediction in patients with MI undergoing PCI. The proposed risk score can be implemented in conjunction with other medical investigations to support treatment decisions and guide clinicians and patients toward individualized healthcare. The advantages of risk tools depend on the local and national healthcare settings. Therefore, it is important to apply this experience to patients with MI undergoing PPCI (43, 44).

### Clinical Implications and Limitations

The findings of this study will provide useful information about the relationship between the bilirubin levels and long-term outcomes in patients with STEMI. We also believe that the predictive effects of the TBil-based score model should be included in intensive therapy and guidelines on risk classifications in the future. This study was mainly limited by its retrospective design and single-center setting; however, a large real-world cohort was enrolled. The potential limitations of our study should be clarified. First, the variables analyzed were baseline covariates, and the treatment strategy was not taken into account. More efforts are needed to optimize this score model so that it may trigger different clinical interventions. Second, subjects were enrolled over a long time span; thus, confounding effects that were derived from progress and improvements in medication and interventional techniques may affect the accuracy of the finding. Thirdly, patients with higher T-Bil was very small which might increase the bias of the study. Therefore, a multicenter study with a short investigation period and larger sample of patients would be preferred.

## Data Availability Statement

The datasets used and/or analyzed during this study are available from the corresponding author on reasonable request. Requests to access these datasets should be directed to Hongbing Yan, hbyanfuwai2018@163.com.

## Ethics Statement

The study was approved by the Ethics Committee of Fuwai Hospital, and all patients provided informed consent for coronary angiography and PPCI.

## Author Contributions

HY and XZ: substantial contributions to conception and design, data acquisition, or data analysis and interpretation. HY, XZ, CL, PZ, ZS, JL, JZ, RC, YW, YC, and HZ: drafting the article or critically revising it for important intellectual content. HY, XZ, CL, PZ, ZS, JL, JZ, RC, YW, YC, and HZ: final approval of the version to be published and agreement to be accountable for all aspects of the work in ensuring that questions related to the accuracy or integrity of the work are appropriately investigated and resolved. All authors: contributed to the article and approved the submitted version.

## Conflict of Interest

The authors declare that the research was conducted in the absence of any commercial or financial relationships that could be construed as a potential conflict of interest.
